# Urinary Reference Values and First Insight into the Urinary Proteome of Captive Giraffes

**DOI:** 10.3390/ani10091696

**Published:** 2020-09-19

**Authors:** Sabrina Fasoli, Giulia Andreani, Francesco Dondi, Enea Ferlizza, Elisa Bellei, Gloria Isani

**Affiliations:** 1 Department of Veterinary Medical Sciences, University of Bologna, Ozzano Emilia, 40064 Bologna, Italy; sabrina.fasoli2@unibo.it (S.F.); f.dondi@unibo.it (F.D.); gloria.isani@unibo.it (G.I.); 2 Department of Experimental, Diagnostic and Specialty Medicine, University of Bologna, 40126 Bologna, Italy; enea.ferlizza2@unibo.it; 3 Department of Surgery, Medicine, Dentistry and Morphological Sciences with Transplant Surgery, Oncology and Regenerative Medicine Relevance, Proteomic Lab, University of Modena and Reggio Emilia, 41124 Modena, Italy; elisa.bellei@unimore.it

**Keywords:** urinalysis, giraffes, biomarkers, electrophoresis, proteomics

## Abstract

**Simple Summary:**

The aims of this study were to determine the urinary reference values and identify the most represented proteins of the urinary proteome in captive giraffes. The reference values for the urine specific gravity, total proteins, creatinine, and urine protein:creatinine ratio, reported as the median, and lower and upper limit, were 1.030 (1006–1.049), 17.58 (4.54–35.31) mg/dL, 154.62 (39.59–357.95) mg/dL, and 0.11 (0.07–0.16), respectively. Giraffes presented a low quantity of urinary proteins, which is in accordance with the data regarding domestic ruminants. In addition to albumin, the most represented urinary proteins were lysozyme and ubiquitin, which are involved in defense mechanisms against microbes.

**Abstract:**

Urinalysis is widely recognized to be a useful tool in routine health investigations, since it can diagnose numerous pathologies. Considering the paucity of knowledge concerning giraffes, urine from 44 giraffes (*Giraffa camelopardalis*) (18 males and 26 females, from 3 months of age to 21 years of age) underwent routine urinalysis, 1D-electrophoresis, and protein identification using mass spectrometry, with the aim of identifying the urinary reference values and the urine proteome. The urine specific gravity (USG), urine total proteins (uTP), urine creatinine (uCr), and urine protein:creatinine ratio (UPC) reference values, reported as the median, and lower limit (LL) and upper limit (UL), were 1.030 (1006–1.049), 17.58 (4.54–35.31) mg/dL, 154.62 (39.59–357.95) mg/dL, and 0.11 (0.07–0.16), respectively. Mass spectrometry, together with electrophoresis, revealed a pattern of common urinary proteins; albumin, lysozyme C, and ubiquitin were the most represented proteins in the giraffe urine. It has been hypothesized that these proteins could act as a defense against microbes. Moreover, in giraffes, urinalysis could be a valid tool for gauging renal function and physiological status changes.

## 1. Introduction

Urinalysis is widely recognized to be a useful diagnostic tool in routine health investigations in veterinary medicine since it can diagnose different metabolic, urinary tract, and systemic diseases [1,2]. In giraffes, the urinary reference values, defined as the interval which contains all of the possible values between and including an upper and lower limit from a reference population [3], have not been defined. Some studies have been carried out on the urinary metabolite concentration and urolithiasis [4,5,6,7] and a preliminary study reported data on urinalysis (e.g., urine specific gravity or dipstick analytes) and proteinuria [8].

Urinary protein patterns can be informative regarding the site of renal damage; for instance, an abundance of proteins with a high and intermediate molecular mass (MM) is indicative of glomerular proteinuria, whereas an abundance of low MM proteins suggests tubular involvement [9]. Therefore, both in animals and humans, specific urinary proteins have been suggested to be clinical biomarkers for obtaining an early diagnosis of renal diseases. A decrease in uromodulin has been suggested to be indicative of tubular disfunction in dogs and cats [10,11,12]. Additionally, an increase in zinc-alpha-2-glycoprotein could be considered as a biomarker for diabetic nephropathy [13] and an increase in albumin, clusterin, or retinol-binding protein has been identified as a possible biomarker of chronic kidney disease or acute kidney injury in humans, dogs, and cats [14,15,16].

The study of the urinary proteome in veterinary medicine is a relatively modern field of research and has been mainly focused on companion and farm animals. In particular, the dog and cat urinary proteome has been investigated using different techniques, as well as those of farm animals such as cow, sheep, goat, and to a lesser extent, horse and pig [17,18]. The literature regarding the proteomics of non-domestic animals is scarce and a few studies have been reported on big cats [19], camels [20], and California sea lions [21].

Since giraffes are liable to have anesthetic-related complications [22], it would be ideal to improve clinical tools and medical examinations which could reduce the stress due to handling as much as possible. The sources of stress are variable, especially for captive animals, and include veterinary examinations, which could affect their welfare [23]. As urine is a biological sample for obtaining important clinical information, and can be collected easily and repeatedly, thus minimizing the stress for giraffes [4,8], the aims of this study were to define the urinary reference values and study the urinary proteome in captive giraffes (*Giraffa camelopardalis*) in an attempt to identify possible biomarkers of health and disease.

## 2. Materials and Methods

### 2.1. Animals and Urine Sampling

One hundred and three urine samples were collected from 44 giraffes (*Giraffa camelopardalis*) living in four different Italian zoos: Parco Zoo Falconara (Falconara Marittima, AN, Italy); ZooSafari Fasanolandia (Fasano, BR, Italy); Giardino Zoologico di Pistoia (PT, Italy); and Safari Ravenna (RA, Italy). Eighteen males and 26 females, from 3 months to 21 years of age, were included in the study; the giraffes belonged to different subspecies (*G. c. rothschildi, reticulata*, and a hybrid). The urine samples were collected from April 2018 to November 2019. During the study period, it was possible to collect urine samples from four pregnant females.

The health status of the giraffes was established on the basis of their clinical examination and history. Giraffes without signs of illness in the 4 weeks before and after the urine sampling, having a good body condition score and no behavior abnormalities, were included in the study.

The giraffes’ diet included alfalfa or hay, fruit (e.g., apple and bananas), vegetables (e.g., carrots and salad), and various dietary supplements (e.g., mixed feeding, bran, corn, fava beans, and cod liver oil). Fresh leaves, grass, and miscellaneous plant branches (e.g., acacia) were given when available and depending on the season.

Animal handling during urine sampling was conducted according to the European Union (EU) Directive 2010/63/EU for animal experiments.

### 2.2. Urinalysis

Urine collection from giraffes was performed during the husbandry activities, without changing the animal’s management, and occurred within 3 hs after the meals between seven and eleven in the morning, under normalized conditions. Using a syringe, immediately after spontaneous voiding, 5 mL of urine were collected individually from the ground or cement in the outdoor areas of all the zoos included in this study, taking only the upper part of the urine, in accordance with similar techniques used in previous research [4,8]. After the collection, individual urine samples were stored and analyzed separately. Each sample, after being transferred with the syringe in a cup, underwent physical and chemical evaluation, and the color and turbidity were evaluated following the guidelines for dogs and cats [1,2]. The urine specific gravity (USG) evaluation was carried out using a refractometer (Giorgio Bormac, 41012 Modena, Italy) and the chemical evaluation using a semi-quantitative dipstick test (KRUUSE VET-10 Urine Strips, J∅RGEN KRUUSE A/S INTERNATIONAL, Langeskov, Denmark). The urine was centrifuged at 1500 *g* for 10 min and a microscopic evaluation of the sediment was carried out under both high (400x) and low fields (100x) [12]. Two drops of urine (~50 µL/drop), including one unstained and one stained with fuchsine solution (Samson Reagenz, Dr. Grogg Chemie AG, Stettlen-Deisswil, CH, Switzerland), were placed on 26 × 76 mm microscope slides (BioSigma, VBS653 Microscope slide, Italy; APTACA Ref.13502, Microscope slides, Italy) and covered with 20 mm^2^ coverslips (PRESTIGE, Micro Cover Glass). The urine supernatants were stored in different aliquots at −20 °C. The urine total proteins (uTP) and creatinine (uCr) were measured using commercial kits (Urinary/CSF Protein, OSR6170, and Creatinine OSR6178, Olympus-Beckman Coulter, Brea, California 92821-6232, USA) on an automated chemistry analyzer (AU 480, Olympus-Beckman Coulter, Brea, CA 92821-6232, USA). The calibration of both methods was carried out using standard materials, in accordance with the manufacturer’s instructions for urine (Urinary/CSF Protein Calibrator; Urine Calibrator; Beckman Coulter, Brea, CA, USA), and the checks were done on a daily basis using a commercially available quality control solution (Liquichek, Urine Chemistry Control, Bio-Rad Laboratories, Irvine, CA, USA). The urine protein:creatinine ratio (UPC) was calculated using the formula UPC = uTP (mg/dL)/uCr (mg/dL).

### 2.3. One-D-Electrophoresis

After thawing and centrifugation at 3000× *g* for 10 min, the supernatants underwent electrophoresis, using sodium-dodecyl-sulfate polyacrylamide gel electrophoresis (SDS-PAGE). The urine proteins were separated using an electrophoresis system (NuPAGE, Thermo Fisher Scientific, Waltham, MA, USA) on precast 4–12% polyacrylamide gel under reducing conditions with MES buffer (2-[*N*-morpholino-ethanesulfonic acid]) (Thermo Fisher Scientific, Waltham, MA, USA) containing SDS. For each sample, 3 μg of proteins was loaded, and the gels were stained with silver nitrate (SilverQuest Thermo Fisher Scientific, Waltham, Massachusetts, USA). After staining, the gels were digitalized using a densitometer (ChemidocMP, BioRad, Hercules, CA, USA) and the pherograms were obtained using commercial software (ImageLab, BioRad, Hercules, CA, USA).

One urine sample from each of the three giraffes was concentrated with spin columns having a molecular weight cut-off of 3 kDa (Vivaspin 500, Sartorius, Goettingen, Germany), following the manufacturer’s instructions. After this process, each sample underwent SDS-PAGE, as previously described, with the exception of a 12% gel, and 15 µg of proteins was loaded and stained with coomassie (Quick Comassie Stain, Protein Ark, Sheffield, UK). The bands were manually excised from the gel and each band underwent mass spectrometry for subsequent protein identification.

### 2.4. Protein Identification by Mass Spectrometry

Protein identification was carried out as previously reported [24,25]. Briefly, the bands underwent in-gel tryptic digestion; the digested dried samples were then re-suspended in 97% Water/3% ACN to which 1% formic acid was added, and were analyzed using an UHPLC-ESI-QExactiveTM (Thermo Fisher Scientific, Reinach, Switzerland) composed of an UltiMate 3000 UHPLC System and an ESI-QExactive Hybrid Quadrupole-OrbitrapTM mass spectrometer (LC-MS/MS-QO System).

Since the giraffe protein database is not annotated, a broader taxonomy, namely “all mammals”, was selected for identification to be based on sequence homology. Protein-identification peak lists were generated using the Mascot search engine (http://mascot.cigs.unimo.it/mascot) against the UniProt database (UniProt.org), specifying the following parameters: Mammalian taxonomy, trypsin enzyme, 1 max missed trypsin cleavage, and Carbamidomethylation (C) as fixed modifications; Deamidated (NQ) and Oxidation (M) as variable modifications; Monoisotopic Mass values; Unrestricted Protein mass; ±10 ppm of peptide mass tolerance; and ±0.02 Da of fragment mass tolerance. Proteins with a score > 80 or identified with at least two significant sequences were selected. The significant threshold in Mascot searches was set to obtain a false discovery rate <5% (5% probability of false matches for each protein with a score above 80). The biological processes, molecular functions, and cellular components of the proteins identified were reported, according to Gene Ontology (GO) and UniProt.

### 2.5. Statistical Analysis

Statistical analysis was carried out using MedCalc Software version 19.3.1 (MedCalc Software Ltd., Ostend, Belgium; https://www.medcalc.org; 2020), according to the guidelines reported for establishing the new reference intervals [3]. Selection of the reference giraffes was carried out following the American Society of Veterinary Clinical Pathology (ASVCP) reference interval guidelines [3]. The giraffes were categorized according to age, i.e., they were considered juvenile (<12 months of age), subadult (>12 months of age, <4 years of age), adult (>4 and <9 years of age), and mature (>9 years of age) [26], as well as sex. The mean values of the repeated measures (*n* = 92) from the same reference giraffes (up to N = 41) were calculated before carrying out the statistical analysis and reference interval determination [27].

The D’Agostino-Pearson test was carried out to test the normal distribution of the data previously displayed graphically using the frequency histograms. *p* > 0.05 was considered indicative of a normal distribution. The outliers were detected using Tukey’s test, by carrying out the test on the mean values (*n* = 41) of the repeated measures from the 41 giraffes. Data not normally distributed were appropriately transformed when needed.

The uTP, uCr, UPC, and USG reference intervals were calculated using the Box-Cox transformation with robust methods (CLSI C28-A3) [3,28]. The 90% confidence intervals (CIs) of the lower limit (LL) and upper limit (UL) were calculated using the Bootstrap method (10,000 iterations; random number seed: 978); the uCr and UPC were back-transformed after the Box-Cox transformation.

Non-parametric tests were used, namely the Mann–Whitney test and the Kruskal–Wallis test, considering sex and age as sources of differences [29]. *p* < 0.05 was considered significant. Regarding SDS-PAGE, the median and range (min-max) values of the number of bands were calculated, and sex and age were considered as sources of difference. The range of protein molecular mass (3–166 kDa) was divided into nine classes (1 = 3–23 kDa; 2 = 23–42 kDa; 3 = 42–62 kDa; 4 = 62–82 kDa; 5 = 82–101 kDa; 6 = 101–121 kDa; 7 = 121–141 kDa; 8 = 141–160 kDa; 9 = 160–180 kDa) by the software (MedCalc) and the relative frequency of each class was calculated for each subgroup. The relative frequency, expressed as a percentage, of each class was calculated by counting the number of bands in each class and dividing the results by the entire number of bands. Graphics were made using Tableau Software (© 2020–2020 TABLEAU SOFTWARE, LLC, A SALESFORCE COMPANY, Seattle, WA, USA).

## 3. Results

### 3.1. Selection of Reference Giraffes and Outlier Identification

Of the 44 giraffes included in the study, three were excluded from the statistical analysis due to pregnancy (N = 2) and one due to the fact that it was in the postpartum period (urine sample collected two weeks after parturition) (N = 1). Another two females were sampled when both pregnant (data excluded from the reference ranges) and not pregnant (data included in the reference ranges).

When Tukey’s test was carried out, no outlier values were identified, except for uTP and UPC, which each revealed one outlier value of 42.95 mg/dL and 0.19, respectively.

### 3.2. Urinalysis

All of the urine samples had a color ranging from light yellow to yellow, while the turbidity was clear/slightly cloudy to cloudy. The pH was between 8.00 and 9.00. The reference interval for USG reported as the mean (lower limit–upper limit) was 1.028 (1.006–1.049). A dipstick test showed negative results in all of the samples analyzed for leukocytes, glucose, ketones, urobilinogen, and bilirubin. Positive results were recorded in 12 giraffes for proteins (30–100 mg/dL), in 5 specimens for erythrocytes (10–50 RBC/µL), and in 12 giraffes for nitrites (trace). The microscopic urine sediment evaluation showed occasional epithelial cells and soil contaminants (pollen, spore, and fibers). Complete urinary data and reference intervals of USG, uTP, uCr, and UPC are reported in Table 1.

The comparisons of the age classes and sex did not reveal significant differences, even though the median values of uTP and uCr in giraffe male urine were higher than in females (uTP = females: 13.70; males: 19.85; uCr = females: 136.05; males: 191.52).

The data for the urinalysis of the pregnant giraffes are reported in Table S1. These values fell into the range established, with the exception of one pregnant female (ID 39).

### 3.3. One-D-Electrophoresis

**Figure 1:**
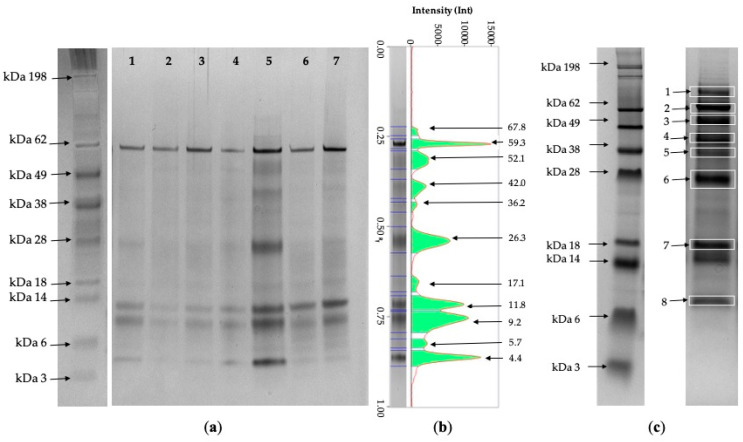
Representative gels of giraffe urine. (**a**) Molecular mass marker (left) and gel (4–12%, silver nitrate staining) (right); lanes 1 and 3–7: female; lane 2: male; (**b**) representative pherogram (lane 5). (**c**) Molecular mass marker (left) and lane from which the bands were excised (right) for protein identification using mass spectrometry. The sample was run on a 12% gel and stained with coomassie. Numbers and arrows indicate the band as in Table 2**.**

Using SDS-PAGE, a pattern of common protein bands was detected in the giraffe urine. The median and range (min-max) of the number of protein bands were 8 and 4–15, respectively. In the majority of samples, the most abundant protein bands had apparent MMs of 64, 50, 42, 34, 25, 14, 10, and less than 6 kDa. The band with the MM at 64 kDa was present in all of the specimens analyzed, as well as the bands at a low MM (14, 10, and less than 6 kDa). On the contrary, the bands at an MM between 18 and 64 kDa and those having an MM higher than 64 kDa only appeared in some samples (Figure 1).

Considering sex and age, there was no difference in the number of protein bands among the groups (*p* > 0.05). Nevertheless, females did not show protein bands higher than 121 kDa, and the relative frequency of those lower than 23 kDa was higher than in males. The bands having an MM lower than 23 kDa were more abundant in giraffes under 9 years of age than in mature giraffes. Conversely, the bands having an MM higher than 101 kDa appeared in mature giraffes (>9 years of age) (Figure S1, Table S2).

Qualitative differences in the urinary proteome were detected between pregnant and not pregnant females. In particular, the pregnant giraffes did not show bands from 101 to 121 kDa, and the relative frequency of those from 23–42 to 121–141 kDa increased (Figure S2).

### 3.4. Protein Identification Using Mass Spectrometry

The proteins identified using mass spectrometry and their functions are reported in Table 2 and Table S3. Fifteen different proteins were identified from eight bands, and two to four different proteins were detected in the majority of them (Table 2). The most represented bands at 64, 14, and less than 6 kDa were albumin, lysozyme C, and ubiquitin, respectively. Other less frequent protein bands were uromodulin, acidic mammalian chitinase, actin cytoplasmic 1, alpha-1B-glycoprotein, apolipoprotein D, cathelicidin-1, clusterin, deoxyribonuclease-1, haptoglobin, lactotransferrin, pepsin A, and zinc-alpha-2-glycoprotein.

**Figure 2:**
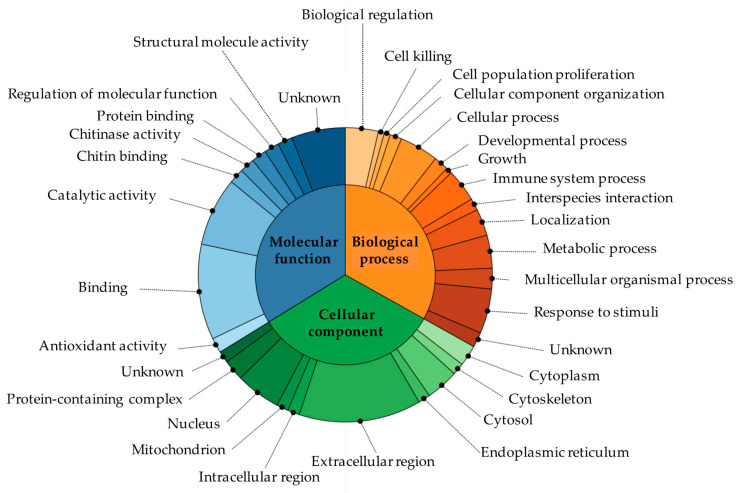
The biological processes, molecular functions, and cellular components of the proteins identified in the urine of the giraffes.

The biological processes, molecular functions, and cellular components related to these proteins are reported according to GO and UniProt in Table S3 and Figure 2. Forty-one percent of the proteins identified were located in the extracellular region (serum albumin, lactotransferrin, acid mammalian chitinase, alpha-1B-glycoprotein, clusterin, zinc-alpha-2-glycoprotein, pepsin A, haptoglobin, deoxyribonuclease-1, apolipoprotein D, and cathelicidin-1); other proteins were present in the nucleus (15%) (clusterin, actin cytoplasmic 1, deoxyribonuclease-1, and ubiquitin), cytoplasm (7%) (acidic mammalian chitinase and ubiquitin), mitochondrion (4%) (clusterin), cytoskeleton (4%) (actin cytoplasmic 1), and endoplasmic reticulum (4%) (apolipoprotein D). The most common molecular function of these proteins was their binding to other molecules (32%) (serum albumin, uromodulin, lactotransferrin, actin cytoplasmic 1, haptoglobin, deoxyribonuclease-1, and apolipoprotein D). Other proteins were enzymes (23%) (lactotransferrin, zinc-alpha-2-glycoprotein, pepsin A, deoxyribonuclease-1, and lysozyme C-2) or had regulatory functions (5%) (lactotransferrin). The proteins identified were involved in several biological processes, e.g., biological regulation, immunity, response to stimuli, cell population proliferation, cell killing, cellular component organization, and cellular and developmental processes (Figure 2).

## 4. Discussion

The physical and chemical examination of the giraffe urine in the present study confirmed data previously reported in the scientific literature, namely the alkaline pH, as reported in cows [17,30] and giraffes [6,8], and the dipstick positivity to proteins, which could be related to the urine alkaline pH and the sampling method [31,32,33]. Moreover, the dipstick positivity to proteins was not confirmed by either the presence of casts in the urine sediment or by the quantitative evaluation of proteinuria (UPC < 0.2). Consequently, it is suggested that a quantitative analytical technique is used instead of a semi-quantity method for determining urine total proteins, due to the high incidence of false positivity in the dipstick test.

The USG values established for giraffes reflected data reported in other herbivorous non-domestic animals, such as Asian elephants [34], Bovidae and Antilocapridae [35], and captive rhinoceros [36], as well as those reported in domestic animals, such as cows [17,30], sheep, and goats [31]. However, the results of this variable should be interpreted carefully and USG should be evaluated repeatedly, since a single sample is not indicative of the urine concentrating ability. In fact, low values, such as 1.003, have been reported in clinically healthy goats [31].

Regarding the presence of outliers, we decided to perform a non-parametric method for determining the reference intervals, since it was less affected by their presence [3]. The reference values established for uTP, uCr, and the UPC in giraffes were in line with data which had previously been reported in the literature for domestic animals [30,37,38]. The giraffe urine presented a low amount of total proteins, as has been reported in other healthy ruminants [17,18]. The interval established for uCr was partially superimposable with previous intervals reported for giraffes [6], and the uCr and the UPC upper limits of the reference interval were slightly lower than those of cattle [30]. However, these data may only be representative of captive giraffes since, in the wild, animals are faced with different environmental and nutritional conditions. This study was carried out on urine collected from animals having ad libitum access to water; this might explain why their urine was less concentrated than expected. The concentrate:hay ratio could influence the urinary values in giraffes [6], and this aspect should also be taken into account.

The comparison between sexes revealed higher values for uTP and uCr in males, albeit not significant, than in females, as in the urine of rat [39]. Urinary creatinine is likely higher in men than in women [40] since the excretion of this metabolite is related to body mass [41]. Accordingly, giraffe males’ average weight is about 1200 kg, while the average weight observed in females is around 800 kg [42].

When considering the specimens excluded from the statistical analysis, their mean values fell into the reference intervals established, except for one specimen (ID 39), for which the UPC mean value was 0.24. This giraffe was a female in her third month of pregnancy and, as reported in healthy women, proteinuria increases during pregnancy [43]. Consequently, it was reasonable to assume that the increase in uTP affected the UPC value. Moreover, the latter mean value was 0.14 when the same female was sampled after pregnancy, which fell into the reference limits established.

The study of the urinary proteome in non-domestic mammals is still in its infancy, despite the biological and clinical importance of these animals as spontaneous models of a wide variety of physiological adaptations or diseases. Mass spectrometry analysis, together with SDS-PAGE, allowed the separation and identification of the 15 most represented urinary proteins. Some of these proteins, e.g., uromodulin, albumin, alpha-1B-glycoprotein, and haptoglobin, are well-known components of the human urinary proteome [44]. Other abundant proteins with a lower MM, such as lysozyme C and ubiquitin, were unexpected. The majority of the proteins identified in giraffes has also previously been reported in the urine of camels [20], cats [12], cows [45], dogs [46,47], and California sea lions [21], suggesting the presence of a common set of proteins in the urine of healthy mammals. However, unlike sea lions, which excrete a urine similar to humans and dogs and dominated by uromodulin, albumin, and protein AMBP [21], the urine of giraffes is characterized by a protein repertoire more similar to that reported in camels. The majority of the urinary proteins in this species were located in the extracellular region and were involved in the immune response, leading the authors to speculate that camels are able to maintain a sterile urinary tract [20].

Uromodulin, which is one of the most abundant proteins in the urine of healthy mammals, is only present in traces in the urine of giraffes, as well as in the urine of cows [17,18], and it can be hypothesized that low amounts of this protein may be characteristic of ruminant urine. Since in vitro uromodulin inhibits the aggregation of calcium oxalate or phosphate crystals, reducing their excretion in the convoluted tubules [48], a possible role of this protein in the formation of these types of crystals, frequently reported in captive giraffes, could be hypothesized [49]. Uromodulin has also been proposed as a pregnancy biomarker in cows, and its decrease is related to tubular dysfunction in dogs and cats [10,11,12,45].

Albumin is usually present in low amounts in the healthy urine of mammals, and its increase has been described in many species as a biomarker of renal dysfunction. In fact, an increase in urinary albumin related to an impairment of glomerular filtration has been reported in dogs and cats with chronic kidney disease (CKD) [11,16].

The presence of acidic mammalian chitinase in giraffe urine is challenging. Despite the absence of chitin, mammals possess two functional chitinases: acidic mammalian chitinase (AMCase) and chitotriosidase (Chit1) [50,51]. It has recently been reported that AMCase can function as a major digestive enzyme which constitutively degrades chitin in the mouse gastrointestinal tract [52]. In addition, AMCase has been shown to prime the immune response against the chitin-containing gastrointestinal nematodes [53]. This protein seems to be a promising biomarker for the diagnosis of sepsis-induced acute kidney injury (AKI), since it was only present in the urine of septic mice with AKI [54]. Since the giraffes included in the present study were clinically healthy and underwent regular antiparasitic treatment, the presence of AMCase in their urine might be considered physiological.

Lysozyme C (muramidase) is a hydrolytic enzyme with bactericidal activity synthesized by monocytes. Due to its low MM, lysozyme is normally filtered by the glomerulus and reabsorbed in the proximal tubule [55]. This protein is one of the most abundant urinary proteins in giraffes and was present in all of the samples analyzed, suggesting a physiological function. The predominance of lysozyme C has also been described in the urine of California sea lions [21]. The authors hypothesized that the abundance of this immune system protein in the urine of sea lions was indicative of high innate immune protection against pathogens. In addition to lysozyme C, the urinary protein pattern of the giraffes revealed the presence of other proteins involved, although in a different manner, in the defense against pathogens, such as acidic mammalian chitinase [51], cathelicidin [56], clusterin [57], lactotransferrin [58], and zinc-alpha-2-glycoprotein [59,60], suggesting their role against pathogens that could colonize the urinary tract.

Ubiquitin, as an essential player in the ubiquitin–proteasome system, is involved in the labeling of damaged or misfolded proteins, and in the physiological degradation of those which are unnecessary. An alteration of this system, and the resulting dysfunctions in protein post-translational modification or catabolism, are associated with different renal diseases [61]. In the kidney, ubiquitination regulates sodium (Na) reabsorption through the electrogenic amiloride-sensitive epithelial Na channel and the sodium chloride (NaCl) cotransporter [62]. In addition, both ubiquitin and actin require particular attention, as they are involved in aquaporin 2 (AQP2) trafficking and modulation [63,64,65]. Actin appears to be involved in the translocation of AQP2 as it is able to directly bind to this water channel [63,64], while E3 ubiquitin ligase CHIP can interact with AQP2 and directly ubiquitylate it in vitro [65]. These data suggested a possible role of ubiquitin and actin in the urine concentration of giraffes. In camels, an overexpression of cytoplasmatic proteins, including actin, has been reported to be an adaptive mechanism for survival with alternative drought-rehydration periods [66], leading to the hypothesis of a similar mechanism in giraffes, which seldom drink water in semi-desert areas [67]. Notably, in comparison with other species, giraffes seem to possess a high concentration and variation of arginine vasopressin (AVP) in their plasma [68]. This hormone regulates AQPs and is involved in the urine concentration and F-actin depolymerization, which is a critical step in AQP2 trafficking [69,70,71], leading to the hypothesis that these complex biological processes and adaptations could have been selected to enable giraffes to survive in their habitat. However, additional studies are needed to corroborate this hypothesis.

Clusterin is a glycoprotein synthesized in the kidney in the early stages of normal development and also in concomitance with various types of acute and chronic injury. Urinary clusterin excretion could be considered to be a useful marker of tubular damage, since it has been observed in both proximal and distal tubules. In children, a decrease in the urinary clusterin concentration has been observed during the postnatal period, suggesting this protein as a possible marker of renal function maturity [72].

Considering the sex-related difference found in the urinary proteome of giraffes, the presence of the bands with a high MM in giraffe males could be explained by considering that, as reported in humans and cows, the urine of males shows specific prostate-origin proteins (e.g., prostatic acid phosphatase, which is a glycoprotein with an MM of approximately 100 kDa), whereas the urine of females shows lipid and carbohydrate metabolism-related proteins [73,74]. Therefore, the different anatomies of the reproductive system could be responsible for some sex-related differences.

The urine proteome of pregnant giraffes was a challenge. It could be speculated that the proteins included between 23 and 42 kDa could refer to haptoglobin, cathelicidin, bovine pregnancy-associated protein, or a group of proteins called pregnancy-associated glycoproteins (PAGs), since they increase during the early days of gestation or only appear when the animal is pregnant [45,75,76,77]. However, additional studies are required to investigate the urine proteome in pregnant giraffes.

## 5. Conclusions

Establishing the urinary reference values allowed knowledge regarding the physiology of giraffes to be improved and can be considered a starting point for clinical applications. The most abundant proteins in the giraffe proteome are involved in the defense against microbes and in urine concentration mechanisms. Interestingly, some of the proteins identified in this study have been used or proposed as biomarkers for renal diseases or pregnancy in other species, which is encouraging for planning additional studies not only to identify possible new urinary biomarkers, but also to confirm, in giraffes, those already validated in other species. Finally, the possibility of assessing the health status of giraffes by means of non-invasive techniques could be useful, even in the wild, for investigating possible factors capable of affecting urinary values.

Giraffes, like other wild animals, present physiological and biochemical adaptations tailored by evolution to cope with specific challenges. Studies should therefore be encouraged because they can represent unconventional models useful for biomarker identification and translational medicine.

## Figures and Tables

**Table 1:** Descriptive statistic and urinary reference intervals.

Variable	N	Mean	Median	SD	LL (90% CI Range)	UL (90% CI Range)
Bil	34	0.0	0.0	0.0	0.0	0.0
Ery (RBC/µL)	34	3	0	9	0	50
Glu (mmol/L)	34	0.0	0.0	0.0	0.0	0.0
Ket (mmol/L)	34	0.0	0.0	0.0	0.0	0.0
Leu (WBC/µL)	34	0.0	0.0	0.0	0.0	0.0
Nit	34	Neg	Neg	-	Neg	Trace
pH	34	9.0	9.0	0.5	8.0	9.0
Pro (mg/dL)	34	30.0	30.0	32.0	0.0	100.0
UBG (µmol/L)	34	0.0	0.0	0.0	0.0	0.0
uCr (mg/dL)	41	145.23	154.62	93.56	39.59 (26.31–61.56)	357.95 (302.62–425.80)
UPC	40	0.11	0.11	0.03	0.07 (0.07–0.08)	0.16 (0.15–0.17)
USG	34	1.028	1.030	0.012	1.006 (1001–1013)	1049 (1044–1053)
uTP (mg/dL)	40	15.78	17.58	8.78	4.54 (3.03–7.09)	35.31 (30.23–40.18)

Bil: Dipstick Urine Bilirubin; Ery: Dipstick Urine Erythrocytes; Glu: Dipstick Urine Glucose; Ket: Dipstick Urine Ketones; Leu: Dipstick Urine Leukocytes; LL: lower limit; N: number of animals included in the statistical analysis; Neg: negative; Nit: Dipstick Urine Nitrate; Pro: Dipstick Urine Proteins; SD: standard deviation; UBG: Dipstick Urine Urobilinogen; uCr: urine creatinine; UL: upper limit; UPC: urine protein:creatinine ratio; USG: urine specific gravity; uTP: urine total proteins.

**Table 2:** Identification of the protein bands in the giraffe urine using mass spectrometry.

N	Database	Accession	Full Protein Name	Species	Score	MM	Mass	Peptides	Pep (sig)	Sequence	Seq (sig)	SC (%)
1	SwissProt	UROM_BOVIN	Uromodulin	*Bos taurus*	602	77	72	55	35	18	14	20%
	SwissProt	TRFL_BOVIN	Lactotransferrin	*Bos taurus*	16	77	80	8	2	8	2	9%
2	SwissProt	ALBU_BOVIN	Serum albumin	*Bos taurus*	1529	64	71	180	104	46	30	50%
	SwissProt	ALBU_SHEEP	Serum albumin	*Ovis aries*	1450	64	71	154	92	42	27	43%
3	SwissProt	CHIA_BOVIN	Acidic mammalian chitinase	*Bos taurus*	196	50	52	16	8	10	5	15%
	SwissProt	A1BG_BOVIN	Alpha-1B-glycoprotein	*Bos taurus*	63	50	54	11	6	5	5	6%
4	SwissProt	CLUS_BOVIN	Clusterin	*Bos taurus*	389	42	51	32	21	15	9	21%
	SwissProt	ZA2G_BOVIN	Zinc-alpha-2-glycoprotein	*Bos taurus*	231	42	34	19	12	9	8	21%
	SwissProt	PEPA_BOVIN	Pepsin A	*Bos taurus*	166	42	40	31	17	8	6	9%
	SwissProt	ACTB_BOVIN	Actin, cytoplasmic 1	*Bos taurus*	50	42	42	11	3	10	3	29%
5	SwissProt	HPT_CAPIB	Haptoglobin	*Capra ibex*	92	34	45	12	6	10	5	18%
	SwissProt	DNAS1_PIG	Deoxyribonuclease-1	*Sus scrofa*	85	34	31	11	4	5	3	19%
	SwissProt	APOD_BOVIN	Apolipoprotein D	*Bos taurus*	64	34	21	12	5	5	3	20%
6	SwissProt	CTHL1_SHEEP	Cathelicidin-1	*Ovis aries*	48	25	18	5	2	4	2	23%
7	SwissProt	LYSC2_BOVIN	Lysozyme C-2	*Bos taurus*	375	18	16	26	16	7	4	51%
	SwissProt	CTHL1_SHEEP	Cathelicidin-1	*Ovis aries*	58	18	18	9	5	7	5	38%
8	SwissProt	UBIQ_CAMDR	Ubiquitin	*Camelus dromedarius*	52	10	8.5	9	3	7	2	80%

N: number of bands identified, as reported in Figure 1c (right). Accession: protein entry name from the UniProt knowledge database; Species: due to the absence of data regarding giraffes in the database, the protein was matched with other mammalian proteins; Score: the highest scores obtained using the Mascot search engine; MM: apparent molecular mass, as predicted by the MM marker in the SDS-PAGE gels and expressed as kDa; Mass: theoretical MM reported in kDa; Peptides: total number of peptides matching the proteins identified; Pep (sig): total number of significant peptides matching the proteins identified; Sequence: total number of distinct sequences matching the proteins identified; Seq (sig): total number of significant distinct sequences matching the proteins identified; SC: sequence coverage.

## Supplementary Materials

The following are available online at https://www.mdpi.com/2076-2615/10/9/1696/s1: Table S1. Mean, median, standard deviation (SD), range (min-max) of dipstick test results, urine specific gravity (USG), urine creatinine (uCr), urine total protein (uTP) and UPC of pregnant giraffes (Number of giraffes = 4; number of samples = 11); Table S2. Relative frequency (percentage) of the 9 MM-classes for all the groups and subgroups studied is reported; Table S3. The function and biological classification of the proteins identified in giraffe urine. The biological processes, molecular functions, and cellular components according to the Gene Ontology (GO) and UniProt databases; Figure S1. Graphical representation of the relative frequency (%) of the molecular mass classes (1 = 3–23 kDa; 2 = 23–42 kDa; 3 = 42–62 kDa; 4 = 62–82 kDa; 5 = 82–101 kDa; 6 = 101–121 kDa; 7 = 121–141 kDa; 8 = 141–160 kDa; 9 = 160–180 kDa) in all of the subgroups studied. Different colors represent the molecular weight classes. The relative frequency of each class was considered as its absolute frequency divided by the total number of classes and expressed as a percentage; Figure S2. Pherograms of a not pregnant female (ID 21), a 3-month pregnant female (ID 39), and a 10-month pregnant female (ID 21). In order to help with visual inspection of the image, the gel zones between 62 and 18 kDa (*) and lower than 18 kDa (**) were divided with dotted lines.
