# Supplementary material for: Urinary Reference Values and First Insight into the Urinary Proteome of Captive Giraffes
Source: Animals (Basel). 2020 Sep 19;10(9):1696. doi: 10.3390/ani10091696 (PMC7552697; doi:10.3390/ani10091696)
Supplement: Supplementary file 1 [file animals-10-01696-s001.zip › sup files/Supplementary Table_Rev-1.docx]

**Table S1.** Mean, median, standard deviation (SD), range (min-max) of dipstick test results, urine specific gravity (USG), urine creatinine (uCr), urine total protein (uTP) and UPC of pregnant giraffes (Number of giraffes = 4; number of samples = 11).

| **Variable** | **Mean** | **Median** | **SD** | **Min** | **Max** |
| --- | --- | --- | --- | --- | --- |
| Bil | 0.0 | 0.0 | 0.0 | 0.0 | 0.0 |
| Ery (RBC/µL) | 1 | 0 | 2 | 0 | 5 |
| Glu (mmol/L) | 0.0 | 0.0 | 0.0 | 0.0 | 0.0 |
| Ket (mmol/L) | 0.0 | 0.0 | 0.0 | 0.0 | 0.0 |
| Leu (WBC/µL) | 0.0 | 0.0 | 0.0 | 0.0 | 0.0 |
| Nit | Neg | Neg | Neg | Neg | Trace |
| pH | 9 | 9 | 0.5 | 8 | 9 |
| Pro (mg/dL) | 0.0 | 0.0 | 15.0 | 0.0 | 30.0 |
| UBG (µmol/L) | 0.1 | 0.1 | 0.1 | 0.1 | 0.1 |
| USG | 1.020 | 1.018 | 0.014 | 1.008 | 1.035 |
| uTP (mg/dL) | 12.93 | 10.48 | 7.33 | 7.51 | 23.25 |
| uCr (mg/dL) | 114.14 | 104.65 | 72.42 | 41.06 | 206.21 |
| UPC | 0.14 | 0.11 | 0.07 | 0.10 | 0.24 |

Bil: Dipstick Urine Bilirubin; Ery: Dipstick Urine Erythrocytes; Glu: Dipstick Urine Glucose; Ket: Dipstick Urine Ketones; Leu: Dipstick Urine Leukocytes; LL: lower limit; N: number of animals included in the statistical analysis; Neg: Negative; Nit: Dipstick Urine Nitrate; Pro: Dipstick Urine Proteins; SD: standard deviation; UBG: Dipstick Urine Urobilinogen; uCr: urine creatinine; UL: upper limit; UPC: urine protein:creatinine ratio; USG: urine specific gravity; uTP: urine total protein


**Table S2.** Relative frequency (percentage) of the 9 MM-classes for all the groups and subgroups studied is reported.

| **kDa classes** | **Interval** | **Relative frequency (%)** | | | | | | | |
| --- | --- | --- | --- | --- | --- | --- | --- | --- | --- |
| *Class* | *kDa* | *Total (N=41)* | *Juvenile (N=4)* | *Subadult (N=12)* | *Adult (N=6)* | *Mature (N=14)* | *Male (N=18)* | *Female (N=23)* | *Pregnant (N=4)* |
| 1 | 3 – 23 | 46.22 | 46.67 | 50.42 | 50.57 | 42.79 | 42.75 | 49.64 | 46.27 |
| 2 | 23 - 42 | 14.92 | 11.67 | 15.97 | 13.79 | 17.31 | 15.24 | 14.60 | 19.40 |
| 3 | 42 - 62 | 19.89 | 18.33 | 15.97 | 22.99 | 21.15 | 19.70 | 20.07 | 19.40 |
| 4 | 62 - 82 | 14.18 | 18.33 | 13.45 | 11.49 | 12.98 | 15.99 | 12.41 | 11.94 |
| 5 | 82 - 101 | 3.87 | 5.00 | 4.20 | 1.15 | 3.37 | 4.83 | 2.92 | 1.49 |
| 6 | 101 - 121 | 0.55 | 0.00 | 0.00 | 0.00 | 1.44 | 0.74 | 0.36 | 0.00 |
| 7 | 121 – 141 | 0.18 | 0.00 | 0.00 | 0.00 | 0.48 | 0.37 | 0.00 | 1.49 |
| 8 | 141 – 160 | 0.00 | 0.00 | 0.00 | 0.00 | 0.00 | 0.00 | 0.00 | 0.00 |
| 9 | 160 – 180 | 0.18 | 0.00 | 0.00 | 0.00 | 0.48 | 0.37 | 0.00 | 0.00 |

**Table S3** The function and biological classification of the proteins identified in giraffe urine. The biological processes, molecular functions and cellular components are reported according to the GO and UniProt databases.

| **Full name of protein** | **Species** | **Biological Process** | **Molecular Function** | **Cellular Component** |
| --- | --- | --- | --- | --- |
| Serum albumin | *Bos taurus* | Cellular process, response to stimuli, biological regulation, localization | Binding | Extracellular region, protein-containing complex |
| Serum albumin | *Ovis aries* | - | Binding | Extracellular region |
| Uromodulin | *Bos taurus* | - | Binding | Intracellular region (secreted) |
| Lactotransferrin | *Bos taurus* | Immune system process, response to stimuli, developmental process, interspecies interactions, biological regulation, cell killing, cellular process, multicellular organismal process | Regulation of molecular function, binding, catalytic activity | Extracellular region, protein-containing complex |
| Acidic mammalian chitinase | *Bos taurus* | Cellular process, immune system process, metabolic process, response to stimuli | Chitinase activity and chitin binding | Extracellular region, cytoplasm |
| Alpha-1B-glycoprotein | *Bos taurus* | - | - | Extracellular region |
| Clusterin | *Bos taurus* | Cellular and metabolic processes, immune system process, cellular component organization, biological regulation, response to stimuli, localization, cell population proliferation | Protein binding | Cytosol, nucleus, mitochondrion, extracellular region, protein-containing complex |
| Zinc-alpha-2-glycoprotein | *Bos taurus* | Immune system process | - | Extracellular region |
| Pepsin A | *Bos taurus* | Metabolic process, multicellular organismal process | Catalytic activity | Extracellular region |
| Actin, cytoplasmic 1 | *Bos taurus* | Cellular process, cellular component organization, localization, response to stimuli, developmental process, biological regulation | Binding, structural molecule activity | Cytoskeleton, cytosol and nucleus |
| Haptoglobin | *Capra ibex* | Response to stimuli, immune system process | Antioxidant activity and binding | Extracellular region |
| Deoxyribonuclease-1 | *Sus scrofa* | Cellular process, metabolic process, immune system process,  biological regulation | Binding, catalytic activity | Nucleus, extracellular region |
| Apolipoprotein D | *Bos taurus* | Developmental process, metabolic process, localization, biological regulation, multicellular organismal process, growth | Binding | Cytosol, endoplasmic reticulum, extracellular region |
| Cathelicidin-1 | *Ovis aries* | Response to stimuli | - | Extracellular region |
| Lysozyme C-2 | *Bos taurus* | Cellular process, response to stimuli, interspecies interaction, multicellular organismal process, metabolic process | Catalytic action | - |
| Ubiquitin | *Camelus dromedarius* | - | - | Nucleus, cytoplasm |
