# Supplementary figures and images for: Urinary Reference Values and First Insight into the Urinary Proteome of Captive Giraffes
Source: Animals (Basel). 2020 Sep 19;10(9):1696. doi: 10.3390/ani10091696 (PMC7552697; doi:10.3390/ani10091696)

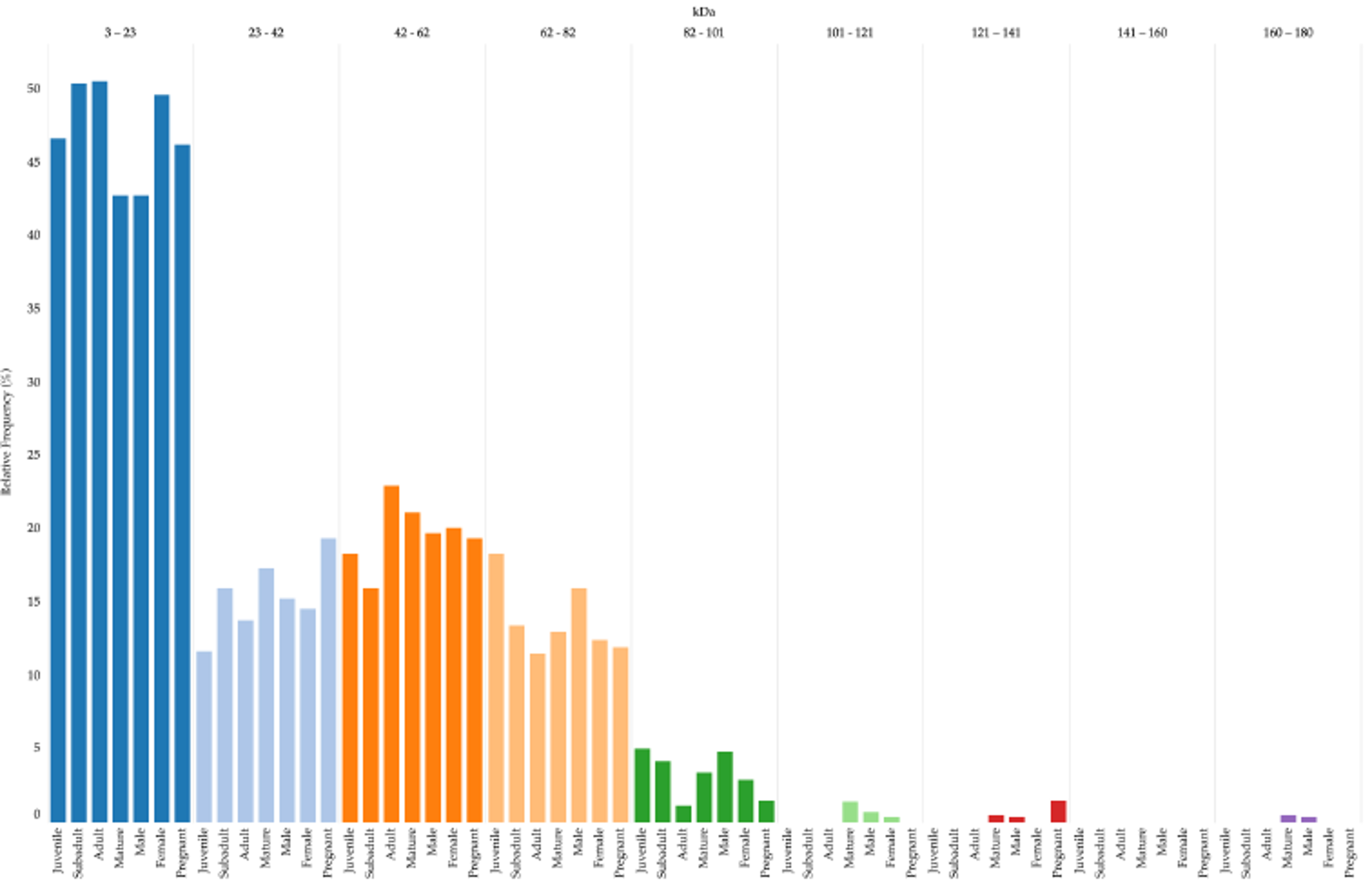

Supplement: Supplementary file 1 [file animals-10-01696-s001.zip › sup files/SuplFig1.jpg]

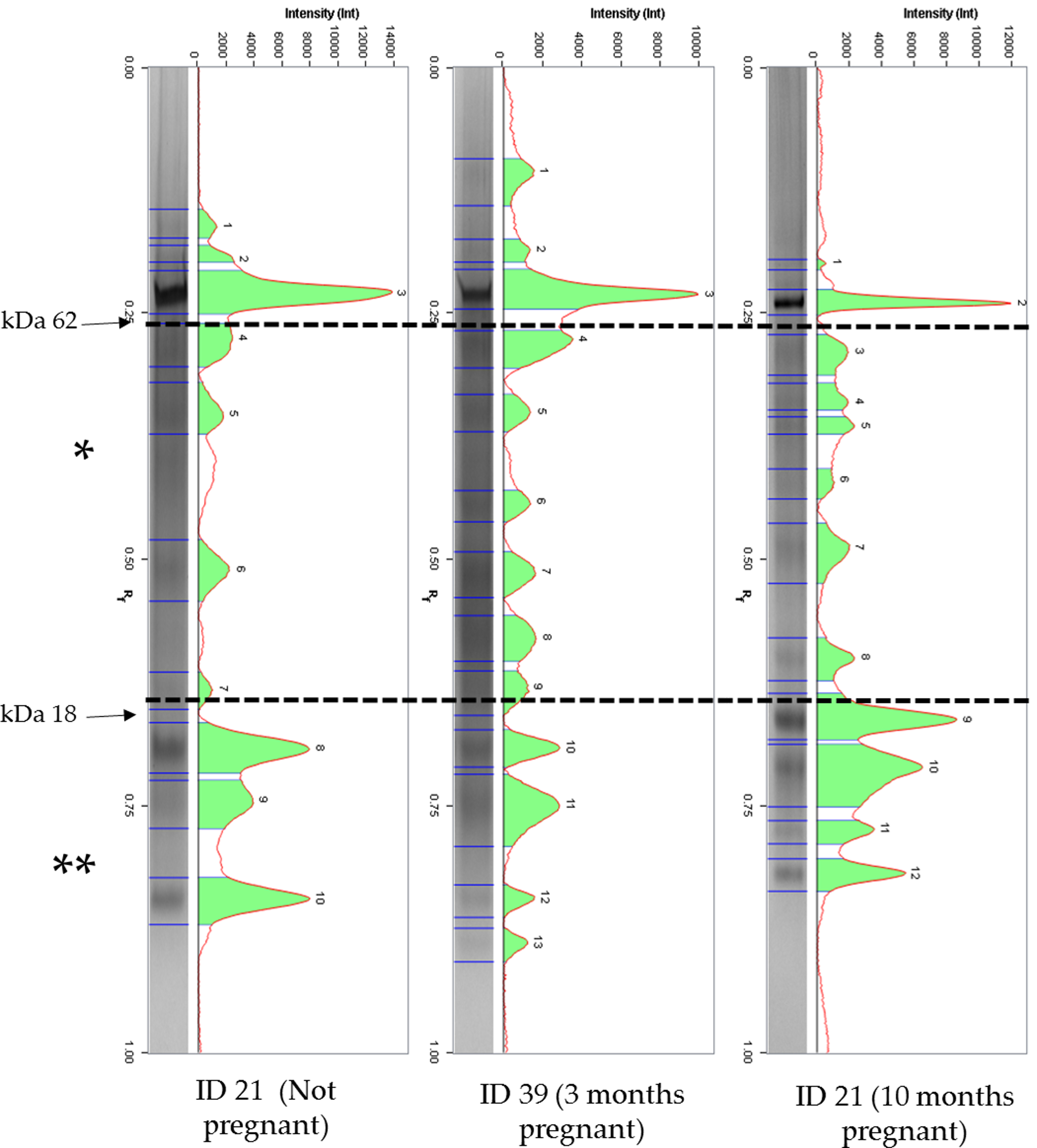

Supplement: Supplementary file 1 [file animals-10-01696-s001.zip › sup files/suplFig2.jpg]
